# The significance of ophthalmological features in diagnosis of thyroid-associated ophthalmopathy

**DOI:** 10.1186/s12938-023-01073-3

**Published:** 2023-02-04

**Authors:** Xiao Huang, Wei Tang, Ya Shen, Linfeng He, Fei Tong, Siyu Liu, Jian Li, Pan Li, Yun Zhang, Xiaoye Ma, Ruili Wei, Weihua Yang

**Affiliations:** 1 Department of Ophthalmology, Changzheng Hospital, Naval Medical University, Shanghai, 200003 China; 2 Department of Endocrinology, Changzheng Hospital, Naval Medical University, Shanghai, 200003 China; 3 Department of Ophthalmology, Naval Medical Center of the PLA, Naval Medical University, Shanghai, 200052 China; 4 The Laboratory of Artificial Intelligence and Bigdata in Ophthalmology, The Affiliated Eye Hospital of Nanjing Medical University, Nanjing, 210004 Jiangsu China

**Keywords:** Thyroid-associated ophthalmopathy, Thyroid function, Ophthalmic images, Auxiliary diagnosis, Graves’ ophthalmopathy

## Abstract

**Background:**

Thyroid-associated ophthalmopathy (TAO) is an autoimmune disorder. It has discriminable appearance. This study was conducted to dig the clinical significance of demographic characteristics and ophthalmologic diagram features in TAO diagnosis and stage/severity evaluation.

**Results:**

We included 320 males and 633 females, with an average age of 41.75 ± 13.75. A majority of TAO patients had hyperthyroidism, and most of them were in the inactive stage and at the moderate level. The thyroid function type, stage and severity were closely associated with hypopsia, eyelid congestion, conjunctival congestion, corneal ulcer, ocular motility disorder, best corrected visual acuity, and extraocular muscle thickening. Using these features, we established different logistic regression models to predict thyroid function subtypes, abnormal thyroid function, stage, and severity, in which the AUC of the ROC curve and accuracies were satisfactory.

**Conclusion:**

Together, TAO subtype, stage and severity can be diagnosed by auxiliary references including demographic factors, symptoms from complains, and image features. These non-invasive indices can be applied in a timely manner in clinical estimating TAO status.

## Background

Thyroid-associated ophthalmopathy (TAO) is an autoimmune disorder. As one of the most vexing problems in endocrinology, it is associated with Graves' disease and can seriously decrease the patients’ quality of life. Almost all patients with Graves' disease have this condition and the majority of them have thyroid involvement [1]. Usually, TAO has discriminable appearance such as exophthalmos, periorbital edema, and eyelid congestion. The etiology of TAO is not completely understood. Known risk factors of TAO include stress, infectious agents, iodine, cigarette smoking, and genes affecting immune function such as HLADR3, CTLA4, PTPN22, CD40, IL-2RA, FCRL3, PPARγ, and IL-23R, as well as genes encoding thyroid-specific proteins like TG [2, 3]. Peribulbar injection of anti-inflammatory drugs or orbital decompression surgery should be recommended for some cases with sight-threatening ocular findings [4, 5]. However, current therapeutic regimens cannot fully restore normal visual function and eye appearance.

According to the changes of thyroid function, TAO can be classified into three subtypes: hyperthyroidism, euthyroidism, and hypothyroidism. When a patient has an impaired thyroid function, spasticity disorders can cause symptoms such as dry eyes, corneal rupture, and periorbital edema. These symptoms can occur in both hyperthyroidism and hypothyroidism cases, but most are related to excess thyroid hormone. Differently, patients with hypothyroidism are more prone to have periorbital edema, while hyperthyroidism cases (as well as those exposed to overdose of thyroid hormone) generally have a staring appearance and retracted eyelids. When thyroid hormone levels increase, patients with Graves’ disease (an autoimmune hyperthyroidism disease) frequently experience cramps [6]. This may be related to the effect of excessive thyroid hormone on the sympathetic nervous system, especially catecholamine excitement (epinephrine or norepinephrine); also, it can be directly caused by anti-thyrotropin receptor antibodies (TRAb) [7]. A lack of thyroid hormone can cause a variety of blood and lymphatic circulation disorders. In particular, fluid retention in the skin causes edema symptoms. However, hyperthyroidism patients may turn to hypothyroidism, and this is frequently accompanied by symptoms (or appearance) of TAO, especially periorbital edema. If thyroid hormone levels return to normal after treatment, these symptoms may be regressed. However, TAO patients with normal thyroid function may also show exophthalmos symptoms due to a previous history of hyperthyroidism which stretches their eyelid muscle fibers. Together, an interesting issue is whether the eye appearance characteristics have diagnostic values in estimating the subtypes of TAO.

According to the EUGOGO standard, TAO can be graded into three severity levels (mild, moderate, and severe) and two stages (active and inactive). Disease severity is the key determinant of indication for therapy; and clinically, a challenge is to recognize the active or inflammatory stage [8]. Similar to thyroid function subtypes, eye appearance or ophthalmic images can present a huge difference between cohorts with different severity levels and grades. For example, orbital positron emission tomography/computed tomography imaging findings can provide references in detecting and grading TAO [9]. Also, amounts of case reports have demonstrated the appearance features of severe or active-stage TAO patients [10–12].

Collectively, in general clinical practice, there are sufficient ophthalmic findings and demographic information, many ophthalmic images can be easily acquired (in a non-invasive and timely manner), which may be informative in auxiliary diagnosis of TAO in aspects of the subtypes (different thyroid function changes), stages, and severity levels. Although there have been different biomarkers for TAO identification [13, 14], it is worth to dig the clinical significance of demographic characteristics and ophthalmologic diagram features in TAO diagnosis and stage/severity evaluation before detection of biochemical indicators. In this context, we conducted a retrospective study based on more than 1000 medical records (953 patients), and several useful regression models were generated for further machine-learning assisted diagnosis of TAO.

## Results

### General information of enrolled subjects

Together, we analyzed the first medical records of 953 cases, including 320 males and 633 females. This sex structure implied that females are more likely to develop TAO than males, which is consistent with known data [7, 15]. The average age was 41.75 ± 13.75 (ranging from 12 to 82). The information of thyroid function types, stages and severity levels is presented in Table 1. Major of TAO patients had hyperthyroidism, and most of them were in the inactive stage and at the moderate level.

**Table 1:** General information of enrolled subjects (*n* = 953)

Characteristics	Value or number	%
Sex		
Male	320	33.6
Female	633	66.4
Age	41.75 ± 13.75 (12–82)	
Thyroid function (*n* = 939)		
Hyperthyroidism	810	86.3
Euthyroidism	112	11.9
Hypothyroidism	17	1.8
Stage (*n* = 937)		
Active	265	28.3
Inactive	672	71.7
Severity (*n* = 923)		
Mild	66	7.2
Moderate	757	82.0
Severe	100	10.8

### Subtype, stage, and severity-associated factors

First, the thyroid function type-associated factors were analyzed. As Table 2 shows, there was a sex difference in thyroid function type distribution. A higher proportion of males showed normal thyroid function than females (*p* < 0.01). Hypopsia was associated with the thyroid function type (but interestingly, only hypopsia of the left eye was significant), that the euthyroidism group had lower percentage of left hypopsia (*p* < 0.01). Also, right eyelid congestion (but not left) showed a correlation with the thyroid function type. Similarly, the euthyroidism group had a significantly lower ratio of right eyelid congestion. Further, these patients with a normal thyroid function had better performance in the best corrected visual acuity of the left eye (but not the right eye), and they had highly significantly less extraocular muscle thickening than the hyperthyroidism and hypothyroidism groups (*p* < 0.01 in all dimensions of extraocular muscle thickening). These parameters were comparable between subgroups with abnormal thyroid function.

**Table 2:** The thyroid function type-associated factors

Factors	Hyper	Eu	Hypo	χ^2^ or F	*P* value
Sex					
Male	259	54	5	11.739	0.003
Female	551	58	12		
Hypopsia (right eye)					
No	658	99	13	3.775	0.151
Yes	152	13	4		
Hypopsia (left eye)					
No	635	99	14	6.147	0.046
Yes	175	13	3		
Eyelid congestion (right eye)					
No	714	104	11	11.602	0.003
Yes	93	8	6		
Eyelid congestion (left eye)					
No	713	101	12	5.523	0.063
Yes	94	11	5		
Best corrected visual acuity (right eye)	0.79 ± 0.32	0.87 ± 0.29	0.74 ± 0.37	2.931	0.054
Best corrected visual acuity (left eye)	0.79 ± 0.32	0.89 ± 0.28	0.77 ± 0.32	4.994	0.007
Extraocular muscle thickening (right eye medial) (mm)	4.51 ± 2.59	3.60 ± 2.49	4.60 ± 2.69	5.761	0.003
Extraocular muscle thickening (right eye lateral) (mm)	3.46 ± 1.77	2.79 ± 1.88	3.20 ± 1.74	6.595	0.001
Extraocular muscle thickening (right eye upper) (mm)	4.78 ± 2.56	3.52 ± 2.43	4.60 ± 2.82	11.167	< 0.001
Extraocular muscle thickening (right eye lower) (mm)	4.51 ± 2.42	3.27 ± 2.34	4.47 ± 2.70	12.214	< 0.001
Extraocular muscle thickening (left eye medial) (mm)	4.70 ± 2.64	3.71 ± 2.56	4.73 ± 2.71	6.541	0.002
Extraocular muscle thickening (left eye lateral) (mm)	3.52 ± 1.86	2.87 ± 1.90	3.40 ± 1.88	5.719	0.003
Extraocular muscle thickening (left eye upper) (mm)	4.79 ± 2.56	3.59 ± 2.53	4.73 ± 2.94	10.180	< 0.001
Extraocular muscle thickening (left eye lower) (mm)	4.55 ± 2.46	3.37 ± 2.39	4.73 ± 2.94	10.748	< 0.001

*Hyper* hyperthyroidism, *Eu* euthyroidism, *Hypo* hypothyroidism

Next, TAO stage-associated factors are presented in Table 3. Males had more cases in the active stage than females, and an elder age was correlated with the active stage. Besides, patients in different stages had distinct features of eyeball pain (both sides), hypopsia (both sides), eyelid congestion (both sides), conjunctival congestion (both sides), corneal ulcer (both sides), ocular motility disorder (both sides), best corrected visual acuity (both sides), and extraocular muscle thickening (all dimensions except the upper and lower extraocular muscle of the right eye). The inactive stage was associated with weaker symptoms of hypopsia, eyelid congestion, conjunctival congestion, corneal ulcer, ocular motility disorder, and better performance in the best corrected visual acuity. However, patients in the inactive stage had a higher extent of extraocular muscle thickening versus those in the active stage.

**Table 3:** The TAO stage-associated factors

Factors	Active	Inactive	χ^2^ or t	*P* value
Sex				
Male	118	199	18.887	< 0.001
Female	147	473		
Age		51.3 ± 12.2	38.1 ± 12.4	14.760
Eyeball pain (right eye)				
No	216	607	13.935	0.001
Evoked	27	34		
Spontaneous	22	31		
Eyeball pain (left eye)				
No	217	612	15.724	< 0.001
Evoked	25	31		
Spontaneous	23	29		
Hypopsia (right eye)				
No	173	595	69.547	0.000
Yes	92	77		
Hypopsia (left eye)				
No	162	584	77.786	< 0.001
Yes	103	88		< 0.001
Eyelid congestion (right eye)				
No	187	640	113.801	< 0.001
Yes	77	30		< 0.001
Eyelid congestion (left eye)				
No	187	637	107.107	< 0.001
Yes	77	33		< 0.001
Conjunctival congestion (right eye)				
No	92	525	168.253	< 0.001
Mild	158	144		
Moderate	6	1		
Severe	8	1		
Conjunctival congestion (left eye)				
No	96	528	167.365	< 0.001
Mild	152	142		
Moderate	7	0		
Severe	9	1		
Corneal ulcer (right eye)				
No	254	666	11.112	0.002
Yes	10	5		
Corneal ulcer (left eye)				
No	250	663	11.788	0.001
Yes	12	7		
Ocular motility disorder (right eye)				
No	57	303	44.422	< 0.001
Yes	206	366		
Ocular motility disorder (left eye)				
No	50	285	45.626	< 0.001
Yes	213	384		
Best corrected visual acuity (right eye)	0.64 ± 0.36	0.86 ± 0.27	8.821	< 0.001
Best corrected visual acuity (left eye)	0.61 ± 0.36	0.87 ± 0.27	10.266	< 0.001
Extraocular muscle thickening (right eye medial) (mm)	3.87 ± 3.31	4.61 ± 2.23	3.222	< 0.001
Extraocular muscle thickening (right eye lateral) (mm)	3.03 ± 2.36	3.51 ± 1.51	2.984	0.003
Extraocular muscle thickening (right eye upper) (mm)	4.35 ± 3.42	4.73 ± 2.17	1.661	0.098
Extraocular muscle thickening (right eye lower) (mm)	4.10 ± 3.22	4.46 ± 2.06	1.614	0.108
Extraocular muscle thickening (left eye medial) (mm)	4.13 ± 3.53	4.76 ± 2.19	2.629	0.009
Extraocular muscle thickening (left eye lateral) (mm)	3.07 ± 2.45	3.59 ± 1.59	3.073	0.002
Extraocular muscle thickening (left eye upper) (mm)	4.31 ± 3.41	4.78 ± 2.18	2.020	0.044
Extraocular muscle thickening (left eye lower) (mm)	4.11 ± 3.31	4.53 ± 2.08	1.878	0.061

Finally, the severity-associated factors are listed in Table 4. Similar to the TAO stage, the severity level was significantly associated with eyeball pain (both sides), hypopsia (both sides), eyelid congestion (both sides), conjunctival congestion (both sides), corneal ulcer (right eye), ocular motility disorder (both sides), best corrected visual acuity (both sides), and extraocular muscle thickening (all dimensions).

**Table 4:** The severity-associated factors

Factors	Mild	Moderate	Severe	χ^2^ or t	*P* value
Sex					
Male	8	254	56	35.405	< 0.001
Female	58	503	44		
Age	31.1 + 8.9	41.2 + 12.9	54.5 + 12.5	74.919	< 0.001
Eyeball pain (right eye)					
No	64	675	70	65.935	0.001
Evoked	1	53	7		
Spontaneous	1	29	23		
Eyeball pain (left eye)					
No	65	676	74	61.708	< 0.001
Evoked	1	51	4		
Spontaneous	0	30	22		
Hypopsia (right eye)					
No	64	657	34	176.324	< 0.001
Yes	2	100	66		
Hypopsia (left eye)					
No	62	637	34	145.057	< 0.001
Yes	4	120	66		
Eyelid congestion (right eye)					
No	64	671	78	13.780	0.001
Yes	2	84	21		
Eyelid congestion (left eye)					
No	64	668	78	13.187	0.001
Yes	2	87	21		
Conjunctival congestion (right eye)					
No	57	514	33	70.685	< 0.001
Mild	9	232	60		
Moderate	0	4	3		
Severe	0	5	4		
Conjunctival congestion (left eye)					
No	56	518	38	58.038	< 0.001
Mild	10	225	56		
Moderate	0	7	1		
Severe	0	5	5		
Corneal ulcer (right eye)					
No	66	746	94	13.918	0.001
Yes	0	9	6		
Corneal ulcer (left eye)					
No	65	740	95	2.168	0.338
Yes	1	14	4		
Ocular motility disorder (right eye)					
No	57	290	7	105.702	< 0.001
Yes	9	462	93		
Ocular motility disorder (left eye)					
No	55	265	7	100.766	< 0.001
Yes	11	488	92		
Best corrected visual acuity (right eye)	0.92 + 0.22	0.82 + 0.3	0.53 + 0.34	40.251	< 0.001
Best corrected visual acuity (left eye)	0.93 + 0.19	0.82 + 0.3	0.47 + 0.33	61.768	< 0.001
Extraocular muscle thickening (right eye medial) (mm)	3.87 + 1.82	4.29 + 2.63	5.46 + 2.67	10.226	< 0.001
Extraocular muscle thickening (right eye lateral) (mm)	3.13 + 1.42	3.29 + 1.81	4.12 + 1.88	9.911	< 0.001
Extraocular muscle thickening (right eye upper) (mm)	3.89 + 1.75	4.51 + 2.58	5.78 + 2.78	13.220	< 0.001
Extraocular muscle thickening (right eye lower) (mm)	3.7 + 1.84	4.25 + 2.46	5.47 + 2.49	13.385	< 0.001
Extraocular muscle thickening (left eye medial) (mm)	3.97 + 1.83	4.49 + 2.69	5.58 + 2.73	9.036	< 0.001
Extraocular muscle thickening (left eye lateral) (mm)	3.17 + 1.47	3.34 + 1.87	4.29 + 2.03	11.645	< 0.001
Extraocular muscle thickening (left eye upper) (mm)	3.9 + 1.82	4.55 + 2.6	5.71 + 2.72	11.338	< 0.001
Extraocular muscle thickening (left eye lower) (mm)	3.7 + 1.77	4.32 + 2.52	5.47 + 2.50	11.945	< 0.001

Together, these features have important values in TAO recognition and evaluation.

### Logistic regression in prediction of subtype, stage and severity

**Fig. 1:**
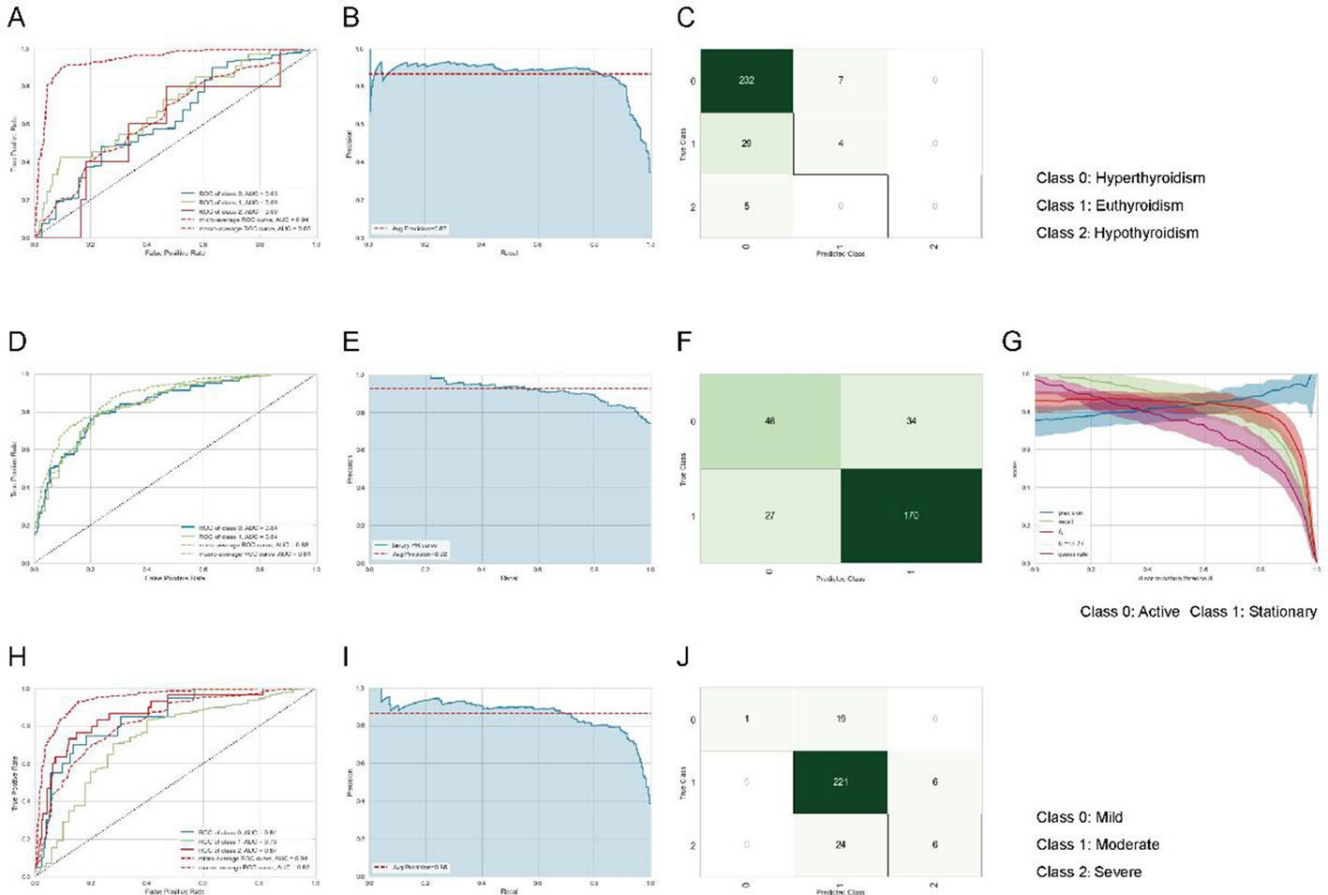
Three logistic regression models for predicting the subtype, stage and severity. These three models were first established and then conducted fine-tuning to maximum the AUC or accuracy using Pycaret. **A** The ROC curve of the thyroid function subtype model (for recognition of hyperthyroidism, euthyroidism, and hypothyroidism). **B** The precision–recall curve for the thyroid function subtype model, with an average precision of 0.87 across different recall levels. **C** The matrix of predicted types and true types in this model. **D** The ROC curve of the two-end logistic regression model of TAO stages, the overall AUC was 0.84 and in the micro-average ROC curve the AUC was 0.88. **E** The precision–recall curve of staging model, with an average precision of 0.92 across different recall levels. **F** The matrix of predicted types and true types, showing an overall accuracy of 0.8. **G** The discrimination threshold plot of this staging model. **H** The three-end logistic regression model of severity, with an AUC of 0.94 in the micro-average ROC curve. **I** The precision–recall curve of this severity model, the average precision was 0.86. **J** The matrix of predicted cases and true cases, with an accuracy of 0.82

Based on above association analysis, all significant auxiliary factors were collected, and the cases with any lacking record of subtype, stage or severity were deleted. Hence, a dataset containing 922 cases was generated, with definite results of the thyroid function type, TAO stage and severity level. Preliminarily, we compared different models according to AUC or accuracy of candidate models and selected the logistic regression model, because it has a satisfactory performance, and the formula of logistic regression is simple and can be drawn out and verified by SPSS. Three models were first established and then conducted fine-tuning to maximize the AUC or accuracy using Pycaret. Three optimized models are presented in Fig. 1. First, the three-end logistic regression of subtype had a good AUC especially in the micro-average ROC curve (*AUC* = 0.94) (Fig. 1A), with an average precision of 0.87 across different recall levels (Fig. 1B). The matrix of predicted and true types is presented in Fig. 1C. In the two-end logistic regression of TAO stages, the overall AUC was 0.84 and in the micro-average ROC curve the AUC was 0.88 (Fig. 1D). In the binary precision–recall curve, the average precision was 0.92 across different recall levels (Fig. 1E). As the matrix of predicted and true types shown, this model had an overall accuracy of 0.8 (Fig. 1F), with a recommended discrimination threshold of 0.27 (Fig. 1G). The three-end logistic regression of severity exhibited an ACU of 0.94 in the micro-average ROC curve (Fig. 1H). In this model, the average precision was 0.86 (Fig. 1I). This model had an accuracy of 0.82 as the matrix of predicted and true types presented (Fig. 1J). Together, these tuned models had a satisfactory efficacy in diagnosis of TAO and evaluating the stage and severity. Moreover, when hyperthyroidism and hypothyroidism were combined into one group (abnormal thyroid function), we used the significant factors (as probed in Table 2) and established a logistic regression for prediction of abnormal thyroid function (Table 5), in which female, elder age, evoked eyeball pain of the left eye, and eyelid congestion were risk factors deserving attention.

**Table 5:** Logistic regression in prediction of abnormal thyroid function

	B	S.E	Wald	P	OR
Sex (female vs. male)	0.890	0.218	16.682	0.000	2.436
Age	0.018	0.008	5.086	0.024	1.019
Eyeball pain (right eye)			14.234	0.001	
Evoked vs. no	− 1.972	0.523	14.234	0.000	0.139
Spontaneous vs. no	− 0.043	0.677	0.004	0.949	0.958
Eyeball pain (left eye)			11.723	0.003	
Evoked vs. no	2.491	0.773	10.384	0.001	12.076
Spontaneous vs. no	−0.750	0.655	1.308	0.253	0.473
Eyelid congestion (right eye)	2.154	0.806	7.139	0.008	8.617
Eyelid congestion (left eye)	− 1.453	0.732	3.944	0.047	0.234
Constant	0.740	0.385	3.690	0.055	2.096

## Discussion

Roughly, the pathogenesis of TAO includes three main phenomena: inflammation of the periorbital soft tissues, overproduction of glycosaminoglycans by orbital fibroblasts, and hyperplasia of adipose tissue. The proliferation of orbital and perimysium fibroblasts produce collagen and glycosaminoglycans in the extracellular matrix. As a consequence, the extraocular muscles swell dramatically [16, 17]. Therefore, many known features can be used in warning of TAO. There have been are imaging studies for diagnosing TAO using CT, magnetic resonance imaging (MRI), ultrasonography (US), and color Doppler imaging (CDI) [18]. The evaluation of extraocular muscle using diffusion-weighted imaging can help detect TAO development [19]. Conjunctival and episcleral inflammation in the extraocular muscles may represent a presenting sign of TAO [20]. In the aspect of severity, some demographic factors have been reported. A British cohort study demonstrated that lower social grade and higher social deprivation, but not ethnicity, had independent, statistically significant association with more severe TAO [21]. Turkish scholars reported that male gender was found as an independent risk factor for severity of TAO [22]. Moreover, an interesting indicator, the ratio of orbital fat to total orbit area, is a useful diagnostic index in mild-to-moderate TAO [23]. Although above studies have revealed the consistent clinical characteristics with the present, very few effective models (focusing on subtypes, stages, and severity levels) have been established using above features.

In the present study, we found that TAO subtype, stage and severity can be predicted by demographic factors including age and gender, symptoms from complains such as eyeball pain and hypopsia, and eye-photo features including eyelid congestion, conjunctival congestion, corneal ulcer, ocular motility disorder, best corrected visual acuity, and extraocular muscle thickening. Our findings are mainly consistent with the consensus of TAO changes, and this work is the first one that combined all associated features and established three models in TAO diagnosis/grading.

In our results, there are some interesting findings never noticed previously. For example, there are side differences in features associated with three TAO outcomes. Hypopsia and best corrected visual acuity in left eye (but not right eye) were associated with thyroid function type. This may be due to a slight limitation of the sample size, for the p values of the right eye just exceeded 0.05 in both hypopsia and best corrected visual acuity. Again, among TAO stage associated factors, upper extraocular muscle thickening in the left eye was less in the active group (*p* = 0.044), but this was not noticed in the right eye (*p* = 0.098). However, the significance of these two indices were both still around 0.05, which implied a weaker indicating effect of the upper extraocular muscle thickening (in comparison with other directions). Moreover, corneal ulcer in the right eye was associated with a severer TAO level, but this trend was not observed in the left eye. In summary, there may be indeed an asymmetry in the indicating roles of ophthalmic symptoms or image features in evaluation of TAO development. However, this asymmetry is to be further confirmed in multi-center observations.

This study has some limitations. For a lack of follow-up data, we mainly retrospectively analyzed the value of ophthalmologic diagrams in the diagnostic period. For this cohort, almost all patients received the orbital decompression treatment. However, no mid-term or long-term follow-up was conducted, hence the prognostic roles of these image features are still unclear. Besides, the relationships between detailed thyroid function indices (such as T3, T4, TSH, TSHR, TRAb, and TSI) and symptoms/image features are not involved in this study (e.g., no linear regression analysis targeting these blood indices has been performed), which restricts the significance of above selected features. Additionally, we found some right/left-side differences in association with the thyroid function, activity, and severity of TAO, and this far, it is still difficult to understand the side influence when predicting these features. But this intriguing finding merits further confirmation and exploration. In addition, the overall AUC and accuracy of our models are still not above 0.9. We have also attempted other models with a little higher AUC or accuracy, such as the Random Forest Classifier, Gradient Boosting Classifier and CatBoost Classifier. All these models cannot provide an ideal prediction, which suggests that more features are needed besides these ophthalmologic diagrams. More potential non-invasive indicators are to be discovered for auxiliary diagnosis and earl evaluation. Finally, microscopic confirmation and proteomic testing can be confirmatory of our conclusion; however, we haven’t collected enough data about the microscopic and proteomic results. The further study can add these data as a support.

## Conclusions

TAO subtype, stage and severity can be predicted by demographic factors including age and gender, symptoms from complains such as eyeball pain and hypopsia, and image features including eyelid congestion, conjunctival congestion, corneal ulcer, ocular motility disorder, best corrected visual acuity, and extraocular muscle thickening. These non-invasive indices are worthy of being collected and applied in a timely manner in clinical practice for TAO detection.

## Methods

### Study population

A total of 953 diagnosed TAO cases admitted in our hospital from 2013 to 2018 were included. The inclusion criteria were as follows: (1) the basic demographic information (such as gender and age) and ophthalmology symptoms (such as eyeball pain, hypopsia, eyelid congestion, ocular motility disorder, upper eyelid late fall, etc.) were recorded; (2) any type of ophthalmology diagrams was documented, including eye appearance or CT image. When a patient had visited more than 1 time, the data of first visit were used. The best corrected visual acuity of both eyes was recorded if available.

With the help of an image recognition artificial intelligence system, following features of both eyes were extracted by two experienced ophthalmologists based on the morphological images: eyelid congestion, conjunctival congestion, corneal ulcer, extraocular muscle thickening (including medial, lateral, upper and lower). The outcomes of these features were first generated by the recognition system and then independently validated by the ophthalmologists. When the opinions of two ophthalmologists differed, they should finally reach an agreement through discussion.

In the aspect of thyroid function, patients were divided into three types according to the diagnosed subtypes: hyperthyroidism, euthyroidism, and hypothyroidism. Further, the combined cohort of hyperthyroidism and hypothyroidism was regarded as the abnormal thyroid function group. Also, different TAO stages were labeled, including the active stage and the inactive stage. Specifically, according to the Clinical Activity Score (CAS) recommended by EUGOGO, a scale with 7 points (each item = 1 point) was used to evaluate the stage: (1) spontaneous eyeball pain; (2) pain on the eyeball or posterior eyeball induced by eyeball rotation; (3) eyelid hyperemia; (4) conjunctival congestion; (5) eyelid edema; (6) bulbar conjunctival edema; (7) inflammation in tear caruncle or fold. A CAS score ≥ 3 points refers to an active stage. The severity was assessed according to EUGOGO standard: Mild (mild eyelid contracture < 2 mm, mild soft tissue involvement, exophthalmos < 3 mm, temporary or no diplopia, and symptoms of corneal exposure are effective for eye moisturizers), moderate (moderate-to-severe, eyelid contracture ≥ 2 mm, moderate or severe soft tissue involvement, eyeball ≥ 3 mm, intermittent or continuous diplopia, mild corneal exposure), and severe (sight-threatening, with thyroid dysfunction, neuropathy and corneal damage).

### Statistical analysis

Categorical data were described by percentages and compared by χ2 test; numeric variables were expressed as mean ± standard deviation (SD) and compared by t-test (between two groups) or one-way ANOVA (among three groups). All data comparison were two-sided, and a p value less than 0.05 was regarded as statistically significant. We mainly focused on three diagnostic outcomes: thyroid function types, TAO stages, and the severity levels. The data were analyzed by SPSS (version 22.0) and the Pycaret python tool (pycaret.org). First, the association between diagnostic outcomes and demographic factors, symptoms and images features were probed by SPSS. The associated factors and each target (outcome label) were further analyzed through Pycaret. Any patient was deleted if this case had an unclear out of thyroid function type, TAO stage, or the severity level. The Pycaret tool preliminarily compared the area under the curve (AUC) of receiver operating characteristic curve (ROC) or accuracy of candidate models (such as random forest classifier, ADA boost classifier, SVM-linear kernel, decision tree classifier, K-Neighbors classifier, and logistic regression). We found that, overall, the logistic regression had a satisfactory power. Besides, the conclusion of the logistic regression can be verified by SPSS and its formula can be clearly drawn out. Therefore, we applied the logistic regression model to show the predictive roles of selected features. Three models were first established and then conducted fine-tuning to maximum the AUC or accuracy. Besides, SPSS was used to establish another logistic regression model of whether a case was in the abnormal thyroid function group based on several thyroid function-associated factors.

## Data Availability

All data generated or analyzed during this study are available from the corresponding author on reasonable request.

## Abbreviations

TAO: Thyroid-associated ophthalmopathy
EUGOGO: European Group on Graves’ Ophthalmopathy
CAS: Clinical Activity Score
TRAb: Thyrotropin receptor antibodies
MRI: Magnetic resonance imaging
US: Ultrasonography
CDI: Color Doppler imaging
SD: Standard deviation
AUC: Area under the curve
ROC: Receiver operating characteristic
